# Incomplete Infection of Secondarily Infected Potato Plants – an Environment Dependent Underestimated Mechanism in Plant Virology

**DOI:** 10.3389/fpls.2017.00074

**Published:** 2017-02-03

**Authors:** Lukas Bertschinger, Lukas Bühler, Brice Dupuis, Brion Duffy, Cesare Gessler, Gregory A. Forbes, Ernst R. Keller, Urs C. Scheidegger, Paul C. Struik

**Affiliations:** ^1^Agroscope, Institute of Plant Production SciencesNyon/Wädenswil, Switzerland; ^2^Programa de Investigaciones en Papa, Instituto Nacional de Investigación Agraria y Agroindustrial, Ministerio de AgriculturaLima, Peru; ^3^Institute for Integrative Biology, Department of Environmental Systems Science, Swiss Federal Institute of Technology (ETZ)Zürich, Switzerland; ^4^School of Life Sciences and Facility Management, Zürich University of Applied SciencesWädenswil, Switzerland; ^5^International Potato CenterLima, Peru; ^6^Institute for Agricultural Science, Department of Environmental Systems Science, Swiss Federal Institute of Technology (ETZ)Zürich, Switzerland; ^7^School of Agriculture, Forest and Food Sciences, Bern University of Applied SciencesZollikofen, Switzerland; ^8^Centre for Crop Systems Analysis, Plant Sciences, Wageningen University and ResearchWageningen, Netherlands

**Keywords:** autoinfection, plant viruses, seed potato systems, seed degeneration, food security, climate change, epigenetics, gene-silencing

## Abstract

The common assumption in potato virus epidemiology is that all daughter tubers produced by plants coming from infected mother tubers (secondary infection) will become infected via systemic translocation of the virus during growth. We hypothesize that depending on the prevalent environmental conditions, only a portion of the daughter tubers of a plant that is secondarily infected by viruses may become infected. To test this hypothesis experimental data from standardized field experiments were produced in three contrasting environments at 112, 3280, and 4000 m a.s.l. in Peru during two growing seasons. In these experiments, the percentage of infected daughter tubers produced by seed tubers that were infected with either potato potexvirus X (PVX), potato Andean mottle comovirus (APMoV), potato potyvirus Y (PVY) (jointly infected with PVX) or potato leafroll luteovirus (PLRV) was determined. Incomplete autoinfection was found in all cases, as the percentage of virus infected daughter tubers harvested from secondarily infected plants was invariably less than 100%, with the lowest percentage of infection being 30%. Changing the growing site to higher altitudes decreased autoinfection for all viruses. Therefore, the assumption of complete autoinfection of secondarily infected plants were rejected, while the hypothesis of environmentally dependent incomplete autoinfection was accepted. The findings help explain the occurrence of traditional seed management practices in the Andes and may help to develop locally adapted seed systems in environments of the world that have no steady access to healthy seed tubers coming from a formally certified seed system. The results obtained almost three decades ago are discussed in light of most recent knowledge on epigenetic regulation of host plant – virus interactions which allow for speculating about the underlying biological principles of the incomplete autoinfection. A research roadmap is proposed for achieving explicit experimental proof for the epigenetic regulation of incomplete autoinfection in the pathosystem under study.

## Introduction

Potato (*Solanum tuberosum* L.) seed tubers used for planting can harbor latent pathogens that subsequently reduce emergence, plant vigor, crop quality and/or yield. Since infected tuber seed is an important source from which these pathogens spread, the proportion of such tubers in a tuber seed lot can accumulate with each consecutive generation, resulting in degeneration of seed quality (Thomas-Sharma et al., 2016). Pathogens of many types are known to infect potato seed, including viroids, viruses, phytoplasmas, bacteria, fungi, and oomycetes (Stevenson, 2001). Historically, seed-borne pathogens have been dealt with in several ways, including selection of better looking mother plants and/or tubers for seed, or acquiring seed from areas known to produce a cleaner product (Young, 1990). For example, in the Andes there is a long-standing practice of producing seed in the cooler highlands and even of moving seed to the highlands to make it more vigorous (Thiele, 1999; De Haan and Thiele, 2003). Propagation systems for pathogen-free seed tubers have been developed that provide growers in industrialized countries with a readily available source of healthy planting stock (Frost et al., 2013). Progress toward developing similar pathogen-free seed propagation systems in the small-holder potato growing regions of the low-income countries has been slow and represents a major production limitation (Thiele, 1999; Thomas-Sharma et al., 2016). Typically, small-holder farmers in low-income countries still produce their own seed or acquire it locally from other farmers, and this seed represents many field generations that have never passed through a process of pathogen elimination.

The Andean region is the origin of the cultivated potato and represents an interesting case for studying seed degeneration. Studies done in traditional Andean potato seed systems over the past 30 years found considerably less than 100% of tubers infected with potato viruses. This was true for viruses that are transmitted by contact (potato potexvirus X (PVX), potato Andean mottle comovirus (APMoV)) or vectored by aphids [potato potyvirus Y (PVY) and potato leafroll luteovirus (PLRV)] (Bertschinger et al., 1990b; Fankhauser, 2000; Haan, 2009; Pérez Barrera et al., 2015). In these studies, PVX, which singly does not cause severe yield loss, was frequently found in relatively high incidence, while PVY and PLRV, which can cause severe yield loss, were generally rare (Bertschinger et al., 1996; Scheidegger et al., 1996; Pérez Barrera et al., 2015).

A potato virus disseminates by infecting during the current growing season a healthy plant subsequently producing infected daughter tubers which disseminate the virus further if they are used as mother tuber, i.e., seed tuber for the plantings of the next season. The terms of primary and secondary infection are commonly used in potato virus epidemiology for the two infection types since many years (see e.g., Beemster, 1967; Hooker, 1982). Primarily virus infected plants produce daughter tubers a proportion of which is usually virus infected (see e.g., Beemster, 1967). If a potato seed tuber (mother tuber) is virus infected, the plant grown from this tuber becomes systemically, so-called secondarily infected, i.e., the pathogen moves through the plant vascular system and eventually reaches, i.e., infects, the daughter tuber tissue, produced by this same plant. While several terms have been used for systemic infection of vegetatively propagated “seed,” such as “autoliberation” for potato viruses (Bertschinger et al., 1990a) or “reversion” (Pacumbaba, 1985), “recovery” (Fargette et al., 1994, 1996) or “self-elimination” (Jennings, 1960; Rossel et al., 1988) for cassava viruses, the common principle behind represents “autoinfection” as defined by Robinson (1976). This is an infection in which the donor (infector) host individual is the same as the recipient (infected) host individual. The term describes a general principle of plant pathogen propagation, which is also applicable to systemic virus infection of the potato daughter tubers produced by an infected mother tuber. The term autoinfection clearly represents in a single word without further descriptive expressions the biological situation of daughter tubers produced by secondarily infected plants, which become themselves systemically infected. This is why we subsequently continue to use this term.

Incomplete systemic infection of daughter tubers from mother tubers infected by mop-top *furovirus* (PMTV) or tobacco rattle *tobravirus* (TRV) was reported some 40 years ago (Cooper et al., 1976; Engsbro, 1976). This was recently confirmed with new data for mop-top *furovirus* (Carnegie et al., 2010; Davey et al., 2014). However, these reports are restricted to few potato-growing zones of the world. There have also been not widely diffused reports that autoinfection may be incomplete also with other more common potato viruses (e.g., Baldauf, 2008). But it is yet commonly held that all daughter tubers of secondarily infected plants will become infected, as explicitely stated in official technical guidance documents (Department of Agriculture and Food, 2016), or implicitly referred to it by not precisely distinguishing between primarily and secondarily infected plants and with what is meant with “infected” (Hooker, 1982; Jayasinghe, 1988).

Considering the limited access to clean-seed systems in the Andes, incidence of systemically infecting seed-borne pathogens such as viruses would be expected to become high over many successive generations with primary and secondary infections and on-site propagation. With 100% autoinfection a modest level of within-season primary disease spread and random seed selection, the overall infection incidence in a tuber progeny should rise successively with every generation to eventually reach high levels, potentially 100%, unless some factors are acting to limit the infection process, or reduce infection incidence once it has occurred. Thus, the low incidence of some major viruses found in Andean potato varieties would support the hypothesis that autoinfection from secondarily infected mother plants to daughter tubers must be incomplete under these circumstances. This incomplete autoinfection could be because not all sprouts or stems from an infected mother tuber will become infected, giving rise to healthy daughter tubers, or because not all daughter tubers on the same infected stem will become infected.

Here, we report on an experimental test of the hypothesis that environment-driven factors may limit autoinfection with four important potato viruses: PVX, APMoV, PVY, and PLRV and we confront the results of these experiments with most recent findings related with virus propagation. These experiments were done in 1987 and 1988 at different altitudes in Peru and were designed to quantify the percentage of daughter tubers becoming infected from mother tubers that were infected by each of the four mentioned viruses. The results clearly indicated that the tuber progeny from infected mother tubers is infected at an incidence much below 100%. The data were initially used to validate a model for temperature-modulated seed degeneration by potato viruses (Bertschinger et al., 1995a,b), which provided implicit evidence for the existence of such phenomenon. However, the findings were not published on their own, primarily because they appeared incompatible with the biological theory at that time. The authors believe that these results should now be made available to the scientific community for two important reasons. First, our knowledge of host-pathogen interactions has greatly advanced indicating how plant defense systems may limit the accumulation and spread of viruses within infected plants (Burgyán, 2006). The above-mentioned results must be re-discussed in view of the nowadays deepened knowledge of host-pathogen interactions with the opportunity to possibly come up with further research questions and sharpen our knowledge in potato virus epidemiology. The second reason is more related to the current on-farm situation. Potato yields are relatively poor in low and middle-low income countries (FAO, 2013) and a major cause of this low productivity is seed degeneration (Fuglie, 2007; Gildemacher et al., 2009; Cromme et al., 2010; Thomas-Sharma et al., 2016). The re-valued results of the above-mentioned studies could contribute to creating an integrated seed health strategy for potato making seed systems more sustainable. To address the constraint of seed degeneration, Thomas-Sharma et al. (2016) recently outlined such a strategy, within which host plant resistance and on-farm disease management play integral roles. They showed that such an approach, including positive selection and the regeneration of degenerated seed, can be used for designing adequate and sustainable seed systems to improve potato productivity in parts of the world that lack access to a continuous supply of healthy seed (Gildemacher et al., 2012; Schulte-Geldermann et al., 2012; Thomas-Sharma et al., 2016).

Thus, there is a need to recheck and confirm the Peruvian data on incomplete autoinfection; a greater understanding of the phenomenon promises to open up new perspectives that could benefit the locally adapted seed systems and eventually increase food security. We therefore first describe, how the Peruvian field data on autoinfection with potato viruses were obtained, document the evidence for the observed, environment-dependent phenomenon of incomplete autoinfection, and subsequently challenge these results with current knowledge in plant virology. Finally, we evaluate our findings and discuss their relevance. We conclude by hypothesizing that tuber infection with potato viruses is limited or even avoided by a mechanism preventing daughter tubers of an infected mother tuber to become infected and that this mechanism is temperature driven.

## Reference Field Data

### Viruses

The experimental work was carried out with potato seed tubers infected with PVX, APMoV, PVY, and PLRV. All PVY infected seed tubers were co-infected with PVX. Damage from PVY is especially high if the plant is co-infected with PVX (Gibson, 1991). The probability for co-infections is high in Peru in view of the extremely high PVX incidence (Monasterios dela Torre, 1966; Bertschinger et al., 1990a,b; Martin-Hernandez and Baulcombe, 2008). For PVY and PVX, virus strain prevalence in the seed tubers utilized for this research was determined at the International Potato Center in Peru (International Potato Center (CIP), 1986, 1988, 1989) in view of the fact that contrasting strains had been identified and for therefore better characterizing the experimental situation. Approximately 90% of the PVY infected tubers were positive for a PVY^N^-like strain, while the remaining 10% were infected by a PVY^O^-like strain. PVX infections belonged to the common O serotype. These strains were the predominant types in the Andean Highlands at the time of the study (Fernandez-Northcote, 1990; Fernandez-Northcote and Lizárraga, 1991).

### Research Sites

Virus-infected tubers were planted in 1987 in three locations representing different agroecological potato growing zones in Peru: Imperial (department: Lima; province: Cañete; district; Nuevo Imperial; latitude: 13°0′ S; longitude: 76°2′ W; elevation: 112 m a.s.l.), Santa Ana (Junin; Huancayo; El Tambo; 12°1′ S; 75°1′ W; 3280 m) and Chicche (Junin; Jauja; Apata; 11°5′ S; 75°2′ W; 4000 m) (**Table 1**). One plot per treatment was planted at each location in 1987, and the experiment was repeated in 1988 with a minor modification in the treatments described below.

**Table 1:** Selected information on experimental sites and crops in Peru.

Name of site	Imperial	Imperial	Sta. Ana	Sta. Ana	Chicche	Chicche
Season	1987	1988	1987/88	1988/89	1987/88	1988/89
Site identity					
Elevation (m)	112	112	3280	3280	4000	4000
Topography	Flat desert belt valley on the Pacific Ocean (up to 800 m)		Bottom of the inter-Andean Mantaro valley (2900–3300 m)		Slope on mountain range between Mantaro valley and jungle	
Agriculture type	Irrigated, commercialized		Rain-fed, partly commercialized		Rain-fed, subsistence-oriented, not commercialized	
Planting-harvest information					
Average daily mean temperature (°C)	18	19	16	12	8	8
Rainfall (mm)	3	1	642	707	^-a^	^-a^
Radiation (MJ/m^2^/day)	9.6^b^	12.1^c^	22.2^d^	19.9^e^	^-a^	^-a^
Planting information^f^					
Week of the year	29	35	49	45	45	41
Average max. temp. (°C)	25	19	26	20	14	20
Average min. temp. (°C)	14	13	6	5	8	3
Harvesting information					
Week of the year	50	4	21	17	21	17
Week after planting	21	21	24	24	28	28
Average max. temp. (°C)	29	27	26	21	13	13
Average min. temp. (°C)	15	19	4	3	2	3

*^a^Incomplete data, or no data available. ^b^CIP Annual Report 1988, average of weeks 18–48, 76°57′ longitude (W), 12°05′ latitude (S), 240 m. ^c^CIP Annual Report 1989, average of weeks 18–48, same site as for ^b^. ^d^CIP Annual Report 1988, average of weeks 48–21, same site as for ^b^. ^e^CIP Annual Report 1989, average of weeks 48–21, same site as for ^b^. ^f^The modern potato cultivar Yungay (*Solanum tuberosum* ssp. *tuberosum* x *S. tuberosum* ssp. *andigena*) was planted.*

### Potato Genotype

The cultivar Yungay (*Solanum tuberosum* ssp. *tuberosum* ×*S. tuberosum* ssp. *andigena*) was chosen because it was widely grown in all zones and susceptible to each of the four viruses used in our study (International Potato Center (CIP), 1986). Infected seed tubers came from on-going experiments in the Mantaro Valley or were multiplied there for the sake of this experiment. Before planting, seed tubers were tested by the enzyme linked immunosorbent assay (ELISA) and selected for single infections with PVX, APMoV and PLRV, and for co-infections of PVX with PVY.

### Field Design, Management, and Data Sampling

Seed tubers (mother tubers), confirmed to be infected according to the testing described below and consequently later giving rise to secondarily infected plants, were planted jointly with healthy seed tubers at the above mentioned research sites in plots with 10 rows, each of 10 m length with 1 m between-row spacing and 0.33 m between-plant spacing, giving a total of 300 plants per plot. Separate plots were planted for each virus species. The infected and the healthy seed tubers were uniformly distributed in each plot of 300 plants (Campbell and Madden, 1990), i.e., healthy and infected seed tubers were evenly distributed in the experimental field plots, while the position of each secondarily infected plant was marked and registered to be able to harvest their tubers for the purpose of this study. The plots were enlarged by extending each row by 2 m at both ends and by adding two border rows adjacent to rows 1 and 10, where on-farm sanitation (weeding and insecticide spraying if needed) was applied. This was to reduce co-infection of daughter tubers with other viruses due to primary infection of the mother plant that could possibly interfere with systemic tuber infection with the viruses under study. Plants received 90-180-180 kg ha^-1^ of N-P_2_O_5_-K_2_O at planting and a further 90 kg N ha^-1^ at hilling. Symptoms of secondarily infected plants were evaluated (presence or absence of symptoms) 6–10 weeks after planting, by checking whether secondarily infected plants were visible or not and whether the within field position of such plants was confirmed. Daily minimum and maximum temperatures were measured 1–2 m above ground and missing data were treated as described by Coakley (1989). The experiments were performed over two growing seasons (1987, 1988), by planting plots with the same experimental design but in a different field at the same site.

### Virus Testing

At harvest of the experimental plots, four randomly selected daughter tubers from each secondarily infected plant were collected. These tubers were stored before testing for virus infection (procedure see below) in paper bags (four tubers per bag, i.e., per plant) placed in wooden trays for 1–2 months after harvest at ambient temperature. Bags were sheltered from wind and rainfall, and protected from tuber moth (*Phthorimaea operculella*) and other storage insects with Sevin^®^(carbaryl dust) as appropriate. From the four tubers harvested per plant and stored as mentioned above, three tubers were tested with ELISA to determine virus presence as described below.

Infection of secondarily infected mother tubers was determined using ELISA on sprout sap after having been stored at 20°C in the dark with more than 60% relative humidity for a minimum of 5 weeks. This insured high virus concentration for subsequent ELISA tests. Sap from tuber sprouts was extracted by grinding jointly two sprouts per tuber in extraction buffer (phosphate buffered saline, 1%; PVP, 0.2%; egg albumen, 0.1%; Tween-20, 0.05%) for ELISA (1:5 w/v) (Gugerli, 1979, 1986). ELISA was performed as described below. Only tubers with proven infection as identified by this procedure were planted to give rise to secondarily infected plants for later measurement of virus infection of daughter tubers.

In preparing daughter tubers for ELISA-testing, tuber dormancy was broken with Rindite (250 ml/m^3^) or Bromoethane (200 ml/m^3^) treatment (Bryan, 1989) for 48 h at 20–25°C in a black box having an internal ventilation system and a capacity of eight trays holding approximately 50 kg of tubers. Trays were maintained afterward for 5 weeks in darkness at 20–25°C and 60–70% relative humidity. Daughter tubers were tested by analyzing tuber sap extracted by using a plant sap extractor (Tecan AG, Hombrechtikon, Switzerland) (Gugerli, 1979, 1986) (ELISA specifications see below). The accuracy of the ELISA assays was ensured by the following procedure, in order to prevent “false negatives” (i.e., tubers with an ELISA result indicating no infection while, in fact, the infection was not detected by ELISA due to limited sensitivity): 100 daughter tubers per Rindite or Bromoethane treatment lot were selected composed by tubers with potentially high likelihood of false negatives. Such a high likelihood was attributed to daughter tubers from secondarily infected plants that were classified by the daughter tuber test as ELISA positive on only one or two daughter tubers per plant but not on all three tested tubers. Confirmation tests were performed on tuber sprout sap or leaf extract from 4 to 5 weeks old plants grown from the selected 100 tubers in an aphid-proof screen house (grow-out tests). If more than 5% of tubers that did not seem to be infected based on the ELISA-test were in fact infected according to the results of the grow-out test, the entire lot was discarded and the data were not used in the subsequent data analyses (occurred in <5% of the tuber batches tested).

### ELISA Procedure

The ELISA test (Clark and Adams, 1977) was performed with polyclonal immunoglobulin (IgG) antisera by the International Potato Center (CIP) for PVX, PVY, and APMoV and with monoclonal antibodies for PLRV (Bioreba AG, Reinach, Switzerland). Polyclonal alkaline phosphatase (AP) conjugates were produced using a standard protocol giving a final concentration of 2580 U AP/0.646 mg IgG/ml. Optimal concentrations for plate coating with IgG and conjugate solution were determined for each antibody by ‘checkerboard’ testing (Converse and Martin, 1990). For coating, polyclonal IgG concentration was applied at 0.66–1.33 μg/ml, depending on the antibody lot. Enzyme conjugates were diluted 750- to 1500-fold. Monoclonal antisera were diluted 1000-fold. Coating IgG, plant extracts and enzyme conjugates were applied at 230, 180, and 200 μl/well, respectively, and incubated at 30°C for 5 h, at 4°C overnight, and at 30°C for 4 h, respectively. Nitrophenyl-phosphate was applied at 1 mg/ml substrate solution, 160 μl/well. Special additives included egg albumin (2% w/v) for extraction and conjugate buffer, and 1 mmol MgCl of conjugate buffer (Gugerli, 1986). Plates were evaluated visually on a backlit screen comparing yellowing intensities of particular wells to the reference wells with buffer, healthy and positive standard extracts (three wells per plate). Absorption values (405 nm) of some plates were measured (Titertek Uniskan II, Flow Laboratories, McLean, VA, USA) to ensure that the tuber treatment and incubation procedures were effective in giving high virus concentrations for easy visual identification of positive wells, and that antisera reactivity remained constant.

### Statistical Analysis

The number of secondarily infected plants considered per season and site varied according to the availability of secondarily infected plants grown from uncut seed tubers. Three sources of variance are to be distinguished for this study: virus, season (year) and site. The percentage of infected daughter tubers was calculated by summing up for each of the four virus classes studied the frequencies of tubers that were determined to be virus infected or uninfected in one particular season and site. Frequencies were then compared using Fisher’s exact test for 2×2 tables. This test enables comparison of low and high frequencies (Fisher, 1990). For each virus, all possible pair-wise comparisons between seasons and sites were made. Based on this analysis, the significance of differences between each frequency was determined and represented with equal or unequal letters in the respective figures and tables. The higher frequencies were compared using χ^2^- tests.

### Evidence of Incomplete Daughter Tuber Infection

**FIGURE 1:**
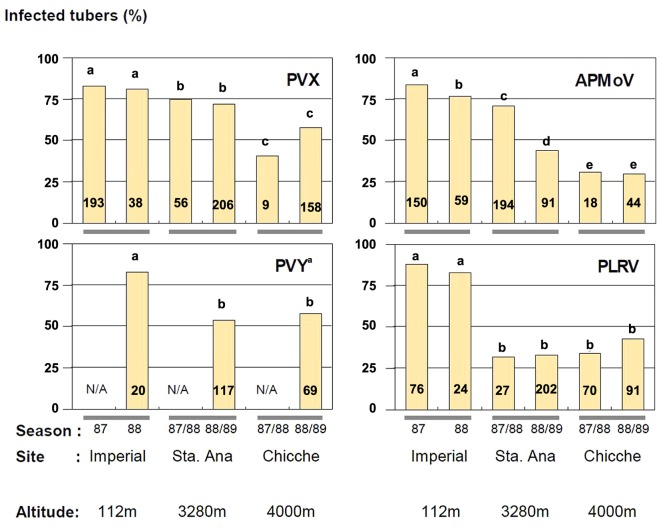
**Tuber infection (% infected tubers) of plants secondarily infected with PVX, APMoV, PVY and PLRV in three agro-ecological zones of Peru.** The cultivar Yungay (*Solanum tuberosum* ssp. *tuberosum* x *S. tuberosum* ssp. *andigena*) was used. Numbers in columns represent the number of analyzed plants. Values within columns having the same letter and corresponding to the same virus are not significantly different in a comparison with Fisher’s exact test (*P* ≤ 0.05). Seed tubers infected with PVY were co-infected with PVX. No data are available for PVY for the seasons 1987 and 1987/88, respectively (N/A). Adapted from Bertschinger (1995).

Incomplete autoinfection was found in all cases, as the incidence of virus infected tubers harvested from secondarily infected plants was invariably less than 100%. The phenomenon was highly variable, depending on virus and location, but with a tendency to have more autoinfection at the lowest altitude. The highest value of autoinfection was obtained with PLRV at 112 m (88%) in 1987 although this was not significantly different from the PLRV percentage observed in 1988 (83%) (**Figure 1**). Also PVX, APMoV, and PVY (together with PVX) were detected in over 80% of tuber infections at 112 m. The lowest percentage of infection (30%) was observed for APMoV at 4000 m in 1988 and 1989 (**Figure 1**).

The effect of the changing environment at the experimental sites was evident for all viruses. The extent of decreasing tuber infection while increasing elevation varied across sites and seasons depending on the virus. For PVX, there were significant differences between all growing sites, while the tuber infection level was in all seasons the same at one particular site. For APMoV, however, there were also significant differences in tuber infection between seasons at one particular site (at 112 and 3280 m) in addition to the differences across sites. Sharp decreases occurred for PVY and PLRV at elevations between 112 and 3280 m, with 27 and 50% average reduction in tuber infections, respectively. Infection did not decrease any further at higher elevations (4000 m). The pattern of symptom expression was correlated to the percentage of tuber infection for PVY, PLRV, and APMoV and was inversely related to the elevation of the potato production plots (**Table 2**). In contrast, PVX symptom expression increased at higher elevation. The highest percentage of secondarily infected plants with symptoms was observed for PLRV at 112 m (99%), while the lowest was observed for PVX also at 112 m (0%). This seems surprising at first instance, since a high systemic infection of tubers suggests high virus titres which – even if only assumingly – might relate to high symptom expression. One explanation for this unexpected result could be that genetic processes governing altered virus replication and translocation as components of ‘resistance’ *sensu* Cooper and Jones (1983) may not be the same as those governing symptom expression, being a component of plant ‘tolerance/sensitivity.’ These genetic processes may respond differently to contrasting environmental conditions in case of PVX. It can be concluded that symptom expression is in general not governed by the same mechanisms as a limitation of autoinfection of daughter tubers of secondarily infected mother plants with potato viruses. Furthermore, symptom evaluation is generally not an appropriate method for detecting virus-infected plants under Peruvian conditions in the case of PVX.

**Table 2:** Symptom expression of plants that were secondarily infected with PVX, APMoV, PVY, and PLRV in three agro-ecological zones of Peru (percent of plants with symptoms, cultivar Yungay ^a,b^).

Plot	Main symptom	Site	Elevation (m)	Season	Number of evaluated plants^c^	Plants with symptoms (%)^d^
PVX	Mosaic	Imperial	112	1987	208	0 b
				1988	206	4 b
		Sta. Ana	3280	1987, 1988	242	17 a
		Chicche	4000	1987, 1988	64	66 c
APMoV	Severe	Imperial	112	1987	196	92 a
	Mosaic			1988	195	63 c
	Mottle	Sta. Ana	3280	1987, 1988	251	79 b
		Chicche	4000	1987, 1988	120	38 d
PVY^e^	Severe	Imperial	112	1987	151	91 a
	Mosaic			1988	124	93 a
	Rugosity	Sta. Ana	3280	1987/88	33	64 b
		Chicche	4000	1987/88	167	31 c
PLRV	Leafroll^f^	Imperial	112	1987	154	99 a
				1988	56	80 b
		Sta. Ana	3280	1987/88	28	75 c
		Chicche	4000	1987/88	132	2 d

*^a^*Solanum tuberosum* ssp. *tuberosum* x *S. tuberosum* ssp. *andigena.*^b^Health status of seed tubers was verified by ELISA. ^c^Symptom evaluation 6–10 weeks after planting. ^d^Numbers which belong to the same virus and which are followed by the same letter are not significantly different in a χ*^2^*-test (*P* ≤ 0.05). ^e^Seed tubers were co-infected with PVX. ^f^Leafroll is a complex of symptoms typical for PLRV (leaf-rolling, especially of lower leaves, yellowing, reduction of the angle between leaf axis and stem, crinkle).*

## Data Reliability

Grow-out tests (see ‘virus testing’ under materials and methods) did not provide evidence for infected tubers that were not detected by the ELISA-testing. There was 99% compliance between the proportion of daughter tubers that were infected with PLRV at 112 m in 1987 (88%) with the proportion of plants with PLRV symptoms in the respective grow-out test. The results of the grow-out tests and the other precautions taken to ensure accurate results (care in tuber storage and confirmation testing and grow-out plots; see ‘reference field data; virus testing’) assured reliability of ELISA-results, avoiding false negatives and assuring that daughter tuber considered to be healthy were indeed healthy and that the calculated autoinfection rates are trustworthy.

### Challenging Experimental Results with Current Knowledge in Plant Virology

Several aspects of current knowledge were reviewed to assess their relevance to reduced autoinfection of daughter tubers from secondarily infected mother plants. These are:

– Host plant genetics;
– Mature plant resistance;
– Environmental factors;
– Anti-viral gene silencing and epigenetic effects.

## Host Plant Genetics

Genetic factors of the host plant were shown to be involved in autoinfection with plant viruses for other pathosystems with autoinfection being an important component of host plant resistance (Fauquet et al., 1988; Fargette et al., 1996). Potato germplasm has not been characterized so far with regard to its genetic variability for autoinfection of secondarily infected plants, neither have the underlying genomics and their interaction with the environment been studied. However, genetic variability for systemic tuber infection in primarily infected plants has been reported, as shown for PLRV (Syller, 1991, 1994, 2003; Difonzo et al., 1994) or PVY (Gibson, 1991). Also, the results of various virus incidence surveys reported for a wide variety of potato genotypes in the Andes (Monasterios dela Torre, 1966; Fankhauser, 2000) point at genetic variability, even if survey data are not a compelling proof for genetic variability with regard to systemic tuber infection factors. Nonetheless, the surveys strongly support the hypothesis that reduced autoinfection may occur in many Andean potato genotypes and that autoinfection may constitute a general mechanism of virus-host pathosystems under Andean conditions. In view of the results of the studies cited, it can be concluded that host plant genetics are a major driver of a limited autoinfection in potato virus pathosystems.

## Mature Plant Resistance

This kind of resistance explains partial tuber infection in primarily infected plants. Beemster (1972) demonstrated for PVY that the older the plant when it becomes (primarily) infected, the more resistant it is in terms of the percentage of tubers that become infected (‘age’ or ‘mature plant’ resistance). Other studies present similar results for PLRV (Barker, 1987; Difonzo et al., 1994; Whitworth et al., 2000) and PVY (Gibson, 1991). This was explained by the fact that the later the plant becomes infected, the less time there is available and the less conducive the conditions are for virus replication, accumulation and systemic translocation inside the plant. Daughter tubers of primarily infected plants might consequently escape infection, be it because of less physiological time for virus multiplication and translocation for reaching tuber tissues or be it because of meristem exclusion mechanisms for virus invasion yet to be proven.

However, both mechanisms of limiting tuber infection can be excluded for secondarily infected plants: First, the period during which viruses can multiply, translocate and invade daughter tubers is not limited in secondarily infected plants, but rather lasts the entire seasons from planting to harvest. Thus, time is not a limiting factor for virus particles to invade tuber tissue. Second, if there were a meristem exclusion mechanism against virus invasion, more developmental time should result in less exclusion while the contrary was observed: at high altitudes, daughter tuber infection was lower than at low altitudes, while there was in fact 3–7 weeks more growth time available at high altitudes for the virus to multiply and translocate systemically (**Table 1**; **Figure 1**).

## Environmental Factors

**FIGURE 2:**
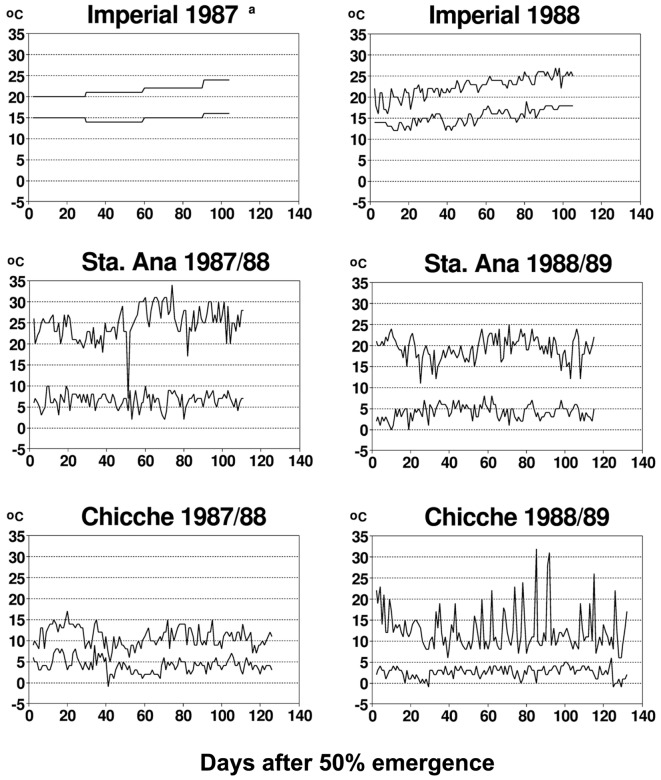
**Minimum and maximum temperatures at experimental sites representing three potato growing zones in Peru.** Only monthly data were available for the Imperial site in 1987. Republished from Bertschinger (1995).

The diurnal temperature variations between the different experimental sites of this study were extremely different (**Figure 2**), providing a range of contrasting environments for studying plant–virus interactions. Temperature has a major and direct impact on virus behavior and the interactions of potato viruses with their host plant or insect vector. Primary infection with PVX or with PVY^N^ or PVY^O^ is altered by temperature (De Bokx and Piron, 1977; Tamada and Harrison, 1981). The same is true for systemic translocation of PVX, PVY^N^ and PVY^O^ (Wislocka, 1982; Adams et al., 1986), replication of PVX, PVY and PLRV (Adams et al., 1986; Gase et al., 1990), resistance of potato genotypes to potato virus M (*Carlaviridae*), PVX, PVY and PLRV infection (Gase et al., 1990), and replication of different strains of PVY (De Bokx and Piron, 1977). Additionally, temperature has been shown to alter cell-to-cell movement of viruses, i.e., the expression of viral encoded transport functions (e.g., for *Potyviridae*) (Zimmern and Hunter, 1983; Dougherty and Carrington, 1988). All these studies provide evidence for the fact that temperature affects plant–virus interaction for primary infections. The effect of temperature on secondary infections is less well-documented. PLRV accumulation in leaves of secondarily infected plants has been shown not to vary with temperature (Tamada and Harrison, 1981; Syller, 1994). However, even if experimental evidence is not yet available in all aspects mentioned above, many of the mechanisms documented by studies addressing primary infections are likely to impact also on systemic virus infection in secondarily infected plants. Interestingly, a temperature-driven model for autoinfection with potato viruses, relating accumulated developmental heat for virus proliferation of daughter tubers of secondarily infected potato plants (Bertschinger et al., 1995a) has been able to reproduce accurately the autoinfection documented in this paper, calibrated by the particular diurnal temperature variations observed in this study (**Figure 2**). Consequently, it can be hypothesized that temperature is a major factor in affecting viral replication and translocation and this may explain why temperature also affected autoinfection in our study.

Low light intensity (irradiance) was shown to significantly increase the severity of mosaic disease caused by potato virus X and potato virus Y (Draper et al., 2002). This was explained by a tentative impact of irradiance on plant–virus interaction in epidermal cells by altering “local acquired resistance” (but not “systemic acquired resistance”) as evidenced with tobacco mosaic tobamovirus (TMV) (Chessin, 1982). However, if applying this knowledge to the symptom expression or autoinfection data from Peru at locations with different levels of irradiance (see **Table 2**), no conclusive pattern arises. Virus-induced leaf lesions, such as a necrosis, are associated with a high respiratory rate of plant cells. Substances involved in respiratory processes accumulate particularly in cells around the necrotic lesions (Yamaguchi, 1960; Parish et al., 1965). Only 60% of dioxygen available at 0 m a.s.l. is available at 4000 m a.s.l., but in our study symptom expression of PVX was observed to be higher at higher altitudes (**Table 2**). Therefore, the scientific data, available so far, on effects of irradiance or dioxygen concentration, are not consistent with the relationship found in this study between the experimental site (i.e., its altitude related with a specific irradiance and dioxygen level) and virus symptom expression or autoinfection.

## Anti-Viral Gene Silencing and Epigenetic Effects

At the time, when the materials presented in this paper were produced, biological theory excluded an active reaction of the plant against the invasion of a virus. However, nowadays, there is scientific evidence for RNA-silencing, a plant mechanism that limits the accumulation and spread of viruses after an infection, and for RNA silencing suppression by plant pathogens: defense, counter-defense, and counter-counter-defense (Pumplin and Voinnet, 2013; Sansregret et al., 2013). It has also been reported, that potato viruses are no exception to this (Voinnet et al., 1999). It is now known whether epigenetics may play a role in potato virus gene silencing (Dalmay et al., 2000). It has been found that temperature affects the regulation of RNA gene silencing and its pathogen regulated suppression. An increased temperature may, e.g., reduce suppression of antiviral gene silencing. But the effect of high temperature on antiviral RNA-silencing and pathogen-mediated suppressor of silencing pathways is not yet fully understood (Qu and Morris, 2005). Epigenetic effects are increasingly understood and potato virus X is one of the model organisms being studied. But many aspects require further elucidation before full comprehension is achieved (Rajeevkumar et al., 2015).

Experimental proof is still lacking that the incomplete autoinfection with potato viruses found in this study is due to temperature-driven epigenetic effects. However, the above-mentioned recent findings in understanding gene-silencing phenomena encourage studying whether these may contribute to observed partial autoinfection with potato viruses reported in our study. In this respect, we consider it promising that a model for temperature-modulated autoinfection (Bertschinger et al., 1995a) was accurately calibrated by the experimental autoinfection data obtained and the prevalent temperature fluctuations presented in this study (see **Figures 1** and **2**).

## Epidemiological and Agronomical Relevance

In traditional seed systems in the Andes, there is no steady supply of healthy seed delivered by a formal seed production system, as there is in industrialized countries. However, partly because of incomplete autoinfection, as shown below, there is in epidemiological terms under certain growth conditions no ultimate necessity of such a supply for preventing low yields because of potato virus epidemics. Assuming random selection of seed tubers from a harvested tuber lot, as well as ambient circumstances where autoinfection is lower than 100% and a low within-season infection rate occurs (i.e., few or no primary infections), e.g., due to low density or minimal prevalence of vectors, the incidence of virus-infected plants will approach zero in consecutive planting generations. This phenomenon may be offset by non-random selection of seed (particularly by a tendency to pick smaller seed which may disproportionately represent virus-infected plants) and/or by high primary infection rates. The interplay of degenerative and regenerative factors could explain why virus incidence in farmers’ seed used in the Andes is often found to be quite low despite the lack of a seed system providing virus-free seed (Bertschinger et al., 1990b; Haan, 2009; Pérez Barrera et al., 2015).

The effect of the three growing sites with contrasting altitudes on autoinfection efficiency in our study also implicitly explains the benefits gained by moving seed from sites at lower to higher elevations, a practice commonly applied by farmers in traditional Andean potato systems (Thiele, 1999). It appears that farmers cleverly made use of the reduced efficiency of autoinfection at higher altitudes to maintain crop productivity and prevent degeneration.

## Crop Management, Positive Selection, and Regeneration of Degenerated Seed

Crop management practices in farmers’ fields differ across agro-ecological zones of Peru represented in this study. For instance, farmers in these different zones may use different planting densities, plant nutrition and other parameters that influence crop performance, affecting tuber size, tuber numbers per plant and other variables. These management differences may also alter plant metabolism, which in turn may impact virus-plant interactions. To control for these potential crop management effects on autoinfection rates, crop management practices were standardized (e.g., uniform mineral fertilization) in all experimental fields of this study.

As noted above, farmers in the Andes have traditionally used altitude to manage and even reverse seed degeneration, but there are additional management options. For example, seed tuber management and crop management (mainly related to managing the maturity resistance), and early harvesting can reduce the level of autoinfection (Struik and Wiersema, 1999). Additionally, a technique to maintain or even improve seed quality has been widely advocated and disseminated by the International Potato Center for application in regions where farmer-saved seed is still dominant, such as Eastern Africa. This technique, referred to as positive selection, involves identifying plants that look healthy at an early stage of the season (e.g., at flowering), which will eventually be harvested for seed at the end of the growing season (Gildemacher et al., 2011, 2012; Schulte-Geldermann et al., 2012; Thomas-Sharma et al., 2016). The positive selection method is simple, cheap and robust, and can easily be adopted by small-holder seed growers or ware growers who plant their own seed. However, it may only work when high plant vigor correlates with low virus infection (i.e., low systemic virus-proliferation of the plant tissue), which might not always be the case, as shown in the study presented in this paper. Schulte-Geldermann et al. (2012) found that a very significant yield increase across agro-ecologies and varieties tested was associated with a reduction in virus incidence for PLPV, PVY, and PVX. However, the reduction in virus incidence could not completely account for the yield increase, suggesting that other factors were also important, such as the interactions among viruses in seed with multiple infections. Gildemacher et al. (2011) also found that yield increase due to positive selection was not always traceable to reduction in virus incidence or other factors.

Applying positive selection might regenerate a degenerated seed stock (i.e., it might reverse degeneration) if consistently and effectively applied across several generations. This is possible if clean seed or almost clean seed is still present in the seed lot that is planted in reasonably high proportions and if plants from those healthy seeds are effectively selected by the positive selection method. However, if this practice is combined with an environment-induced reduced efficiency of autoinfection as described in this paper, the sanitation effect of such practices should be significant and most of all agronomically most relevant. Although little literature data is available on long-term effects of positive selection, some research projects are currently ongoing in Uganda and elsewhere in Africa to assess to what extent regeneration is feasible and to what extent techniques such as positive selection can stop or delay autoinfection under severe or mild aphid pressure, starting with high-quality or low-quality seed lots (e.g., Priegnitz et al., 2014). In view of the data presented in this paper, the environment dependent contribution of incomplete autoinfection to seed regeneration must be taken into account for improving seed and seed systems. In light of the presented results, costly seed certification systems as found in the industrialized countries, which are based on epidemics with a rapidly increasing share of infected tubers due to the assumption of 100% autoinfection of secondarily infected plants, may be replaced by locally adapted seed multiplication systems for controlling virus incidence in seed.

## Conclusion and a Research Roadmap

The commonly held theory in potato virus epidemiology that all daughter tubers of a seed-tuber infected mother plant (secondary infection) are systemically infected, is rejected by this study. The hypothesis that potato tuber infection by viruses (in the present study PVX, APMoV, PVY, and PLRV) may be limited or even avoided by a mechanism preventing daughter tubers of an infected mother tuber to become infected, is clearly supported by the presented field data.

This seemingly simple theory has great practical consequences, since costly, formal seed potato systems in industrialized countries in temperate climates are based on this assumption, supported by evidence under these climates. However, these formal systems have also been applied (albeit unsuccessfully) in low-income countries with different climatic conditions and traditional, informal seed systems.

Based on an extensive literature review, it can be concluded that reduced autoinfection is seemingly environment-dependent and most likely temperature-driven. Recent findings regarding the epigenetic regulation of plant virus – host interactions suggest that temperature-driven anti-viral gene silencing may explain the described phenomenon observed in Peruvian potato fields. However, this remains yet to be experimentally proven for the specific potato virus pathosystems addressed in this paper.

While the traditional epidemiological theory of potato viruses must be reconsidered in the light of these findings, the latter open up new perspectives for improving food security by contributing to environmentally adapted effective and sustainable seed potato systems and also for breeding purposes. These results should be of particular benefit in potato growing zones where a continuous supply of healthy seed produced by a formal certification has, to date, been unsuccessful. Also, the combination of the findings with those developed in other studies for improving seed systems, e.g., implementing on-farm practices such as positive selection, will contribute to the design of a farmer-friendly package of management practices that further improve the productivity of the crop.

The discovered environmental dependency of incomplete autoinfection is also of great importance in view of the challenges experienced by climate change. A change in plant virus epidemics may be forecasted for global potato growing zones, which could inform policy designed to mitigate potentially negative effects of climate change.

Explicit experimental proof of the hypothesized temperature-driven RNA-silencing in the addressed potato virus pathosystems would facilitate exploitation of the principle of incomplete autoinfection. The genetic base and inheritance of incomplete autoinfection with potato viruses should be characterized, as well as its epigenetic dimension. Quantifying temperature-driven regulation of the observed phenomenon would facilitate efforts to improve seed quality and food security under varying environmental conditions.

## Author Contributions

Conceived and designed the experiments: LBe, CG, EK, and US. Performed the experiments and analyzed data: LBe. Discussed and validated the results in view of new research findings and scientific evidence and wrote the paper: LBe, LBü, BDuf, BDup, GF, US, and PS.

## Conflict of Interest Statement

The authors declare that the research was conducted in the absence of any commercial or financial relationships that could be construed as a potential conflict of interest.
